# Supplementary material for: Ultrastructure of the nebenkern during spermatogenesis in the praying mantid Hierodula membranacea
Source: PLoS One. 2023 Jul 27;18(7):e0285073. doi: 10.1371/journal.pone.0285073 (PMC10374135; doi:10.1371/journal.pone.0285073)
Supplement: Attachment — Submitted filename: Response to Reviewers.docx [file pone.0285073.s011.docx]

Dear Sir or Madam:

Below we respond to the Editor/Reviewers.

Editor Comments:

1. The abstract is very simple, the main conclusion is not included in the manuscript.

We agree that the previous abstract could be improved and we have added text to the abstract, including the main conclusion.

2. Improve the quality of some of the figures, refer to the reviewer's comments.

We were not exactly sure which figures the reviewers referred to when criticizing the quality. We suspect that during the upload the figures were sampled down. We have re-created the figures with 600dpi to improve visibility and quality.

3. What is the main function of “nebenkern”?

Thanks for this remark. Indeed, we did not introduce the function of the nebenkern sufficiently before. This definition has now been added to the introduction of the paper.

4. Novelty of this study should be pointed in the "Conclusion" part.

We excuse if our original text was not precisely in bringing the novelty across to the readers. We have added a paragraph to the end of the manuscript concluding the paper and discussing the novelty.

Reviewer Comments:

Reviewer #1:

1. The Abstract of this manuscript is too sample to attract readers. And the main point of this manuscript is not summarized in the Abstract.

We agree that the previous abstract could be improved and we have added text to the abstract, including the main conclusion.

2. Although the new methods were used to study the ultrastructure of sperm development, the topic of this manuscript was insufficient of innovation. Besides, the quality of all figures was not high, and several of them were blurry.

The topic of this manuscript was a new description of the ultrastructure of spermatogenesis in a taxon that had not previously been examined. For us this fulfills the criterion of being novel. It also used new methods (high-pressure freezing of testes tissue and electron tomography to investigate larger volumes ultrastructurally) and revealed a previously-unobserved structure (zipper-like membrane of the nebenkern). We have added more text to emphasize these points. In addition, we have re-generated the figures with 600dpi to make them less “blurry” and provide the original TIF images in an open-access repository.

3. The function of the nebenkern during spermatogenesis was not mentioned in this manuscript, so the scientific value and research significance of this study was not outstanding.

We have added more information related to the function of the nebenkern to multiple sections of the paper.

4. Most of the refs cited in this paper are too old. More recent studies should be cited. And the format of the refs should be checked and revised carefully.

We dispute this comment as inappropriate. No scientific reference is too old, especially with a structure studied as long as the nebenkern. Our references go from the 1830s to the 2020s because they appropriately cite material relevant to this research.

We thank the reviewer for pointing out that there are errors in our references section—we have edited that section for our resubmission.

Reviewer #2:

1. In the manuscript of review versions, you don’t number the line in the whole paper. I hope you add the line number if you submit your manuscript in the future.

We excuse for making the review process harder without the possibility to address problems with the correct line numbers. We have added them now.

2. In the section of “Movie legends -Movie 4”, “Stitched” should be changed into “Stitched”.

We appreciate the Reviewer’s noticing this typographical error! It has been fixed now.

3. In the section of “Figure legends”, there are many abbreviations of proper nouns, the full names appear just need appear once, you can use these abbreviations after the second.

The abbreviations in the legends refer to the abbreviations used in the figures to properly describe the contents of the cell. For readability of the legends we think using the proper words instead of the abbreviations are superior.

4. It will be better to change your alpha code form lowercase to capital letter in your figures, because there are both capital letter to code your figures in “Figure legends” and manuscript.

Thanks for pointing this out! These changes have been made

5. Why you used the development stage of subadult male praying mantid as your object in this research, but not male praying mantid in other development stage? What advantages of the subadult mantid?

Thanks for pointing out this fact that needed to be clarified. We have added some sentences to the Methods explaining our use of subadult males. That developmental stage is the only stage in which spermatogenesis can be observed—younger male mantids have testes containing pre-meiotic cells and adult mantids have only mature sperm cells in their testes.

6. In your article, you focused on the “spermatids at different stage of development”, but I don’t know clearly the staging criteria. In addition, I think there is a little chaos in your “spermatocyte” and “spermatid”, you should distinguish the two stages more clearly.

We have been very careful to use the term spermatocyte to describe cells that are after the spermatogonial stage but prior to the full completion of cytokinesis of meiosis II. Spermatids are cells that have completed meiosis II.

7. Can you explain the differences of “axonemal microtubules” with “microtubules” for me?

Axonemal microtubules are simply microtubules associated with the axoneme. However, when investigating the region of more mature sperm cells, we found microtubules not associated with axonemes. As we were not sure which function they serve in particular we called them only “microtubules”.

8. I want to know more about the function of nebenkern during spermatogenesis in insects, I mean you should supply more information of nebenkern in your “Introduction” section. For example, its formation process, composition, function in reproduction and so on. This part is a little poor in your manuscript now.

Thanks for pointing this out. We have added text describing this better to the manuscript.

9. In Figure 3, you mentioned that you get seven serial semi-thick (300 nm) sections, but there are just four sections you show?

We think here is a misunderstanding. The structure we show in Figure 3 was created by reconstructing, stitching and segmenting 7 single electron tomograms. This stitched volume of about 2.1 µm was then used for creating the images in the figure.

In total, we examined 4 regions by electron tomography in this paper: prophase region (single 300 nm section; Fig.1), the nebenkern (seven 300 nm sections; Fig.3), the nucleus in the mature sperm region (single 300 nm section; Fig.4D) and the axonemes in the mature sperm region (single 300 nm section; Fig.4E).

10. Why you want to examine the microtubules in the 3D structure of nebenkern? In your opinion, if the cytoskeleton participates in the formation of nebenkern, I think it is necessary to examine the dynamical changes of microfilament during this process.

In this initial survey, we observed and documented the location of microtubules with reference to the nebenkern. As the cells were extracted from wild-type animals we had no possibility to investigate the dynamic nature of microtubules in developing sperm cells. Future work will be necessary to look at changes during the process but we agree that this is an interesting direction to explore.

Sincerely,

Gunar Fabig
